# Respiratory Models Reveal DNA Damage Response Modulation by Merkel Cell Polyomavirus

**DOI:** 10.3390/ijms27083449

**Published:** 2026-04-12

**Authors:** Sara Passerini, Marta De Angelis, Sara Messina, Daniela Scribano, Cecilia Ambrosi, Ugo Moens, Lucia Nencioni, Valeria Pietropaolo

**Affiliations:** 1 Department of Public Health and Infectious Diseases, Sapienza University, 00185 Rome, Italy; sara.messina@uniroma1.it (S.M.); daniela.scribano@uniroma1.it (D.S.); 2 Department of Public Health and Infectious Diseases, Laboratory Affiliated to Istituto Pasteur Italia-Fondazione Cenci Bolognetti, Sapienza University, 00185 Rome, Italy; marta.deangelis@uniroma1.it (M.D.A.); lucia.nencioni@uniroma1.it (L.N.); 3 Laboratory of Virology, Department of Molecular Medicine, Sapienza University, 00185 Rome, Italy; 4 Laboratory of Microbiology, IRCCS San Raffaele Roma, 00143 Rome, Italy; cecilia.ambrosi@uniroma5.it; 5 Department of Medical Biology, Faculty of Health Sciences, UiT-The Arctic University of Norway, 9037 Tromsø, Norway; ugo.moens58@gmail.com

**Keywords:** Merkel Cell Polyomavirus, DNA Damage Response, respiratory models, transcriptional level, oncogenesis

## Abstract

Merkel Cell Polyomavirus is an oncogenic virus associated with Merkel Cell Carcinoma (MCC). However, considering viral detection in respiratory specimens and similarities between MCC and neuroendocrine lung cancer, its plausible role in the respiratory tract is disputed. MCPyV-mediated oncogenesis involves viral antigens interfering with host signaling as a DNA Damage Response (DDR). In the current study, respiratory models, including lung cancer cell lines (A549 and H1299), and non-malignant bronchial systems (HBEC-KT and a 2D ALI model) were used to investigate DDR genes’ expression following MCPyV infection. Once the capability to support viral replication and transcription was assessed using qPCR and RT-qPCR, respectively, the mRNA levels of DDR genes, including *ATM*, *ATR*, *Chk1*, *Chk2*, *H2AX*, *Rad51*, *p53* and *p21*, were examined. Our findings showed MCPyV replication in all cellular systems, as proven by the detection of viral DNA and transcripts. Viral infection induced an overexpression of DDR genes, suggesting a role of the virus in manipulating DDR to favor its replication or contribute to tumor progression. These preliminary results provide in vitro models for studying the interplay between MCPyV and DDR within malignant and non-malignant contexts across the respiratory tract, laying the basis for future research exploring the clinical relevance of DDR activation in virus-driven malignancies.

## 1. Introduction

Merkel Cell Polyomavirus (MCPyV) is a small DNA oncogenic virus responsible for Merkel Cell Carcinoma (MCC), a rare and highly aggressive skin malignancy [1,2]. MCPyV possesses a circular double-stranded DNA genome organized into early and late coding regions separated by a Non-Coding Control Region (NCCR) [3]. The early region expresses tumor antigens, referred to as Large T (LT) and small t (sT), implicated in viral replication and transcription and in cellular transformation. The late region, instead, produces the structural proteins, Viral Protein (VP) 1 and 2 and two microRNAs (miRNAs), involved in modulating early gene expression [4,5].

**Figure 1:**
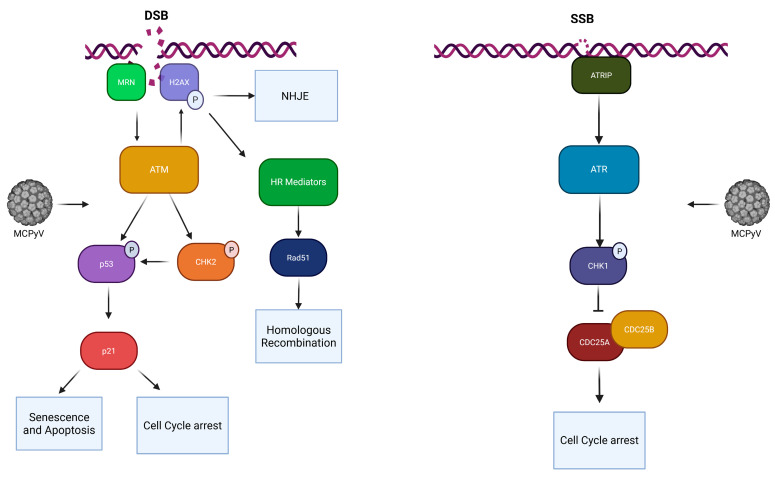
Overview of the DDR signaling pathway. P = phosphorylation. Created in BioRender. Pietropaolo, V. (2026) https://BioRender.com/k1v9qpb (accessed on 8 March 2026).

While primary MCPyV infection is generally persistent and asymptomatic, viral persistence and the dysregulation of cellular pathways may be critical for virus-driven oncogenesis [4,6]. Among the host pathways targeted by MCPyV, DNA Damage Response (DDR) plays a central role in maintaining genomic integrity and was found to be modulated by several DNA viruses to promote viral replication and persistence [7]. DDR is a complex array of signaling pathways that collectively maintain the integrity of the genome [8]. Among the key DDR signaling components, there are protein kinases, ataxia telangiectasia mutated (ATM) and Rad3-related (ATR), activated by double-stranded breaks (DSBs) and single-stranded DNA lesions, respectively [9]. ATM and ATR phosphorylate multiple downstream substrates, including Chk2 and Chk1, as well as the histone variant H2AX, a well-established marker of DNA damage [8,9]. In addition, ATM, ATR, and their downstream effector kinases mediate post-translational modifications of p53, thereby promoting its stabilization and transcriptional activity. Activated p53 induces the expression of target genes such as p21, which plays a key role in the regulation of cell cycle arrest, apoptosis, and senescence [10] (Figure 1).

Previous research demonstrated MCPyV’s capability to interfere with key regulators of DDR, thus modulating cell cycle progression and DNA repair [11,12]. In addition, our recent study reported an overexpression of DDR genes such as *ATM*, *ATR*, *Chk1* and *Chk2* in MCPyV-positive MCC, suggesting the plausible role of the virus in stimulating the transcriptional activation of these genes [13]. However, how viral infection may influence DDR-related transcriptional responses remains largely unexplored. A major limitation in the field is the lack of a well-defined and universally accepted cellular model to investigate MCPyV infection, hindering mechanistic studies of virus–host interactions [14].

Although MCC arises from the skin, accumulating evidence supports MCPyV as a respiratory pathogen. In fact, MCPyV DNA was frequently detected in respiratory specimens, and epidemiological data suggest viral transmission through the respiratory route [15,16]. Moreover, respiratory epithelial cells have been found to support viral replication, indicating the airway epithelium as a plausible site of viral entry and replication [17,18,19].

An additional link between MCPyV and the respiratory tract emerges from the histological similarities between MCC and lung cancers [20,21]. Furthermore, MCPyV was found integrated in a subset of Non-Small-Cell Lung Cancer (NSCLC) specimens, suggesting a plausible role of the virus in the development and progression of this type of tumor [22].

Taken together, these findings support that MCPyV may contribute to oncogenic processes beyond the skin and underscore the relevance of respiratory epithelial models to explore MCPyV interaction with host signaling.

Based on this background, this preliminary study aimed to investigate the expression of DDR genes following MCPyV infection in four respiratory cell lines. Understanding how MCPyV modulates host DDR signaling at the mRNA level may provide new insights into viral persistence mechanisms and early events contributing to oncogenesis, thereby enabling the development of effective strategies to prevent and treat virus-driven malignancies.

## 2. Results

### 2.1. MCPyV DNA Replication in Transfected Cells

Transfection experiments were performed using two malignant cell lines, A549 and H1299, and two non-malignant systems, the bronchial epithelial HBEC-KT and the 2D ALI model, derived from NHBE bronchial cell line. Viral DNA was detected in cell fractions from 2 to 8 d.p.t. Moreover, except for ALI cultures, MCPyV load was also reported in SPNTs, which were then used in subsequent infection experiments (Table 1).

### 2.2. MCPyV DNA Replication in Infected Cells

To assess the permissivity of the employed cell lines, infection experiments were performed using SPNTs obtained from transfection. For ALI cells, SPNTs collected from bronchial HBEC-KT cells were used.

**Figure 2:**
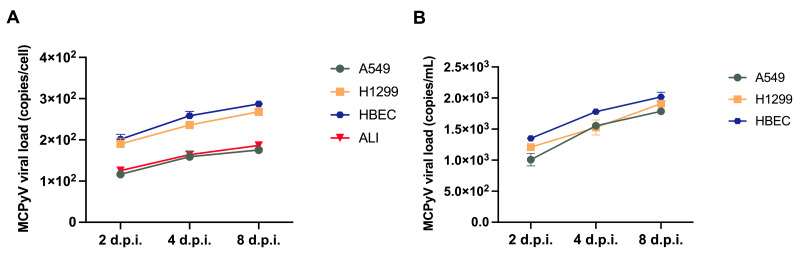
Replication studies of MCPyV in cells (**A**) and supernatants (**B**) during infection experiments. MCPyV replication was assessed at selected sampling times, from 2 to 8 days. MCPyV DNA was quantified using qPCR and is expressed as copies/cell for cells and copies/mL for supernatants. Data are expressed as the means of three independent experiments, and error bars indicate standard deviations.

MCPyV DNA was detected in infected cells, with an increase in viral load during the time course experiment (Table 1, Figure 2A). Moreover, when comparing MCPyV replication in cell fractions and SPNTs recovered from monolayer cell cultures, a similar trend was reported (Figure 2B). Conversely, when analyzing viral load in apical and basal medium from ALI cultures, no traces of viral DNA were detected.

### 2.3. Early and Late Genes’ Transcription

**Figure 3:**
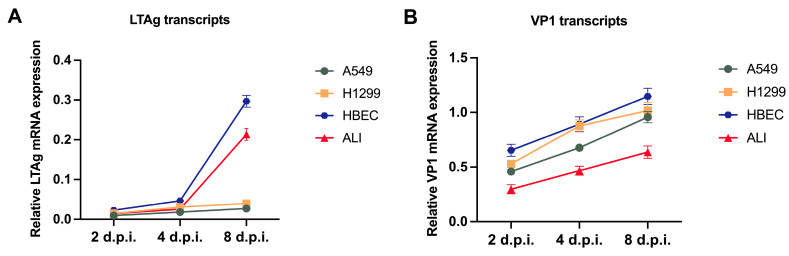
Relative expression of MCPyV *LTAg* and *VP1* transcripts in infected cells at selected sampling times, from 2 to 8 d.p.i. Following RNA extraction and reverse transcription, *LTAg* and *VP1* genes’ expression was measured using qPCR. Relative mRNA levels were normalized to *GAPDH* and expressed as 2^−ΔCt^. Data are represented as the mean of three independent experiments, and error bars represent standard deviations.

The efficiency of viral replication was further assessed by evaluating MCPyV early and late genes’ expression in infected cells. Transcription analysis revealed that both *LTAg* and *VP1* were readily detectable at 2 d.p.i. Although different amounts of early and late transcripts were reported, both *LTAg* and *VP1* expression showed an increase during the sampling period (Figure 3).

### 2.4. mRNA Levels of DDR Genes

Once we assessed that the employed cell lines were permissive to the virus and thus able to support viral replication, the DDR pathway was investigated at the mRNA level, focusing on *ATM*, *ATR*, *Chk1*, *Chk2*, *H2AX*, *Rad51*, *p53* and *p21*.

**Figure 4:**
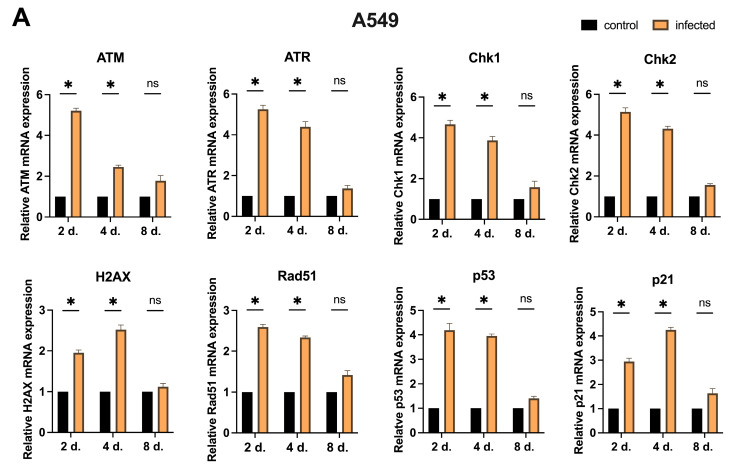

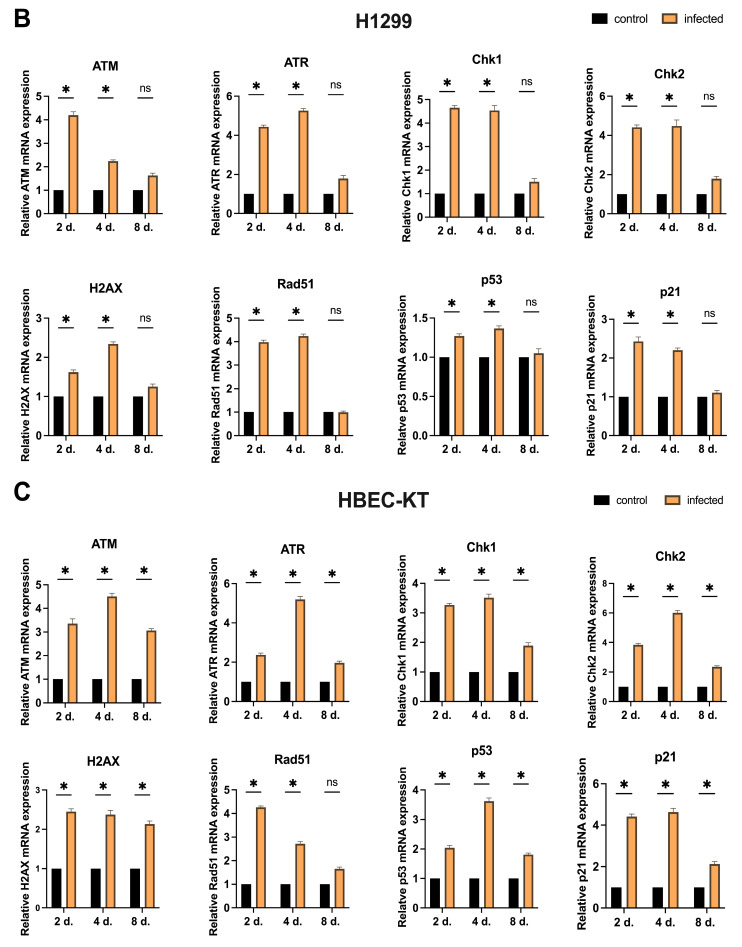

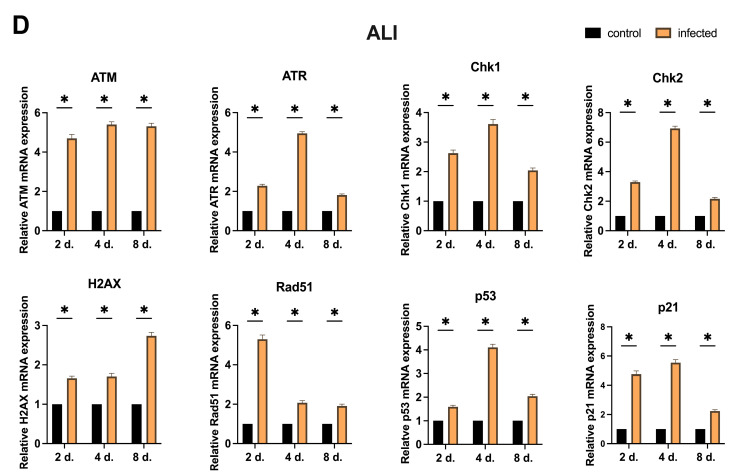
Relative expression of DDR genes (*ATM*, *ATR*, *Chk1*, *Chk2*, *H2AX*, *Rad51*, *p53* and *p21*) in controls and MCPyV-infected cells at selected sampling times from 2 to 8 days (Panel (**A**), A549; Panel (**B**), H1299; Panel (**C**), HBEC-KT; Panel (**D**), ALI). Genes’ expression was measured using RT-qPCR. Relative mRNA levels were normalized against the housekeeping *GAPDH* gene and expressed as 2^−ΔΔCt^. Data are reported as the means of three independent experiments, and error bars indicate standard deviations. Genes’ expression values followed a normal distribution and were compared using Student *t*-test with Bonferroni correction. * *p* < 0.05; ns: not significant.

When comparing infected cells with controls, a dynamic modulation of DDR genes was observed upon MCPyV infection despite different timing and mRNA levels across cell models. Overall, early post-infection time-points revealed a significant overexpression (*p* < 0.05) of DDR genes (Figure 4).

By mid-time-points (4 d.p.i.), some DDR factors maintained an elevated expression, while others began to decline. At later stages of infection (8 d.p.i.), most DDR genes returned toward baseline levels in A549 and H1299 cells, whereas, in non-malignant models (HBEC-KT and ALI cultures), they remained overexpressed (*p* < 0.05)

Notably, although H1299 cells harbor a partial deletion of the *p53* gene and do not express detectable p53 protein, measurable levels of *p53* transcripts were detected and were significantly modulated by viral infection at early time-points (*p* < 0.05) (Figure 4B).

In addition, while for most cell models *H2AX* revealed higher mRNA levels at early stages, in ALI cultures, it reached a maximum at 8 d.p.i., indicating a delayed accumulation of DNA damage (Figure 4D). The comparison of transcript levels of DDR genes within the different cell lines is shown in Figure 4 (A549, Panel A; H1299, Panel B; HBEC-KT, Panel C; ALI, Panel D).

## 3. Discussion

Although it is frequently misrecognized, the primary objective of viruses remains to complete their life cycle rather than promote carcinogenesis [23]. In this framework, previous research revealed that some oncogenic viruses, including MCPyV, manipulate the DDR pathway, plausibly to ensure the replication of its genome. However, as a side effect, this disruption may lead to an abnormal viral replication as well as an accumulation of unrepaired genetic lesions in host DNA, thereby contributing to oncogenic transformation [11,13].

Given the capacity of the virus to manipulate DDR pathways, the current study explored whether similar mechanisms are engaged in respiratory epithelial contexts, in light of evidence linking MCPyV to respiratory infection and lung tumorigenesis [22]. To achieve this goal, multiple respiratory cell models were employed to compare virus–host DDR interplay across distinct cellular contexts. Specifically, lung cancer cells A549 and H1299, previously used to investigate MCPyV infectious process, were used to resemble a tumoral context [17,18,19,24]. In addition, HBEC-KT was utilized to examine MCPyV’s effect on DDR in a non-malignant context, as well as ALI cultures, which provide a more physiological and relevant representation of the airway epithelial barrier, recognized as a plausible site of viral exposure and persistence [15]. Our findings showed that MCPyV is capable of initiating and sustaining viral genome replication in all the examined cellular systems, as proven by the detection of viral DNA in cell fractions and supernatants upon infection experiments. The results from qPCR assays reported an increase in viral load during the time course experiment in all models, although with different amounts. This observation likely reflects differences in cellular differentiation and the proliferative state among tumoral and non-malignant contexts, which may affect replication efficiency [25]. MCPyV replication in respiratory cell models was further confirmed through the detection of early and late transcripts in cell fractions. Notably, consistent with previous studies, transcript analysis revealed that *VP1* mRNA levels were higher than *LT* mRNA, even at early time-points [17]. Overall, these findings support the possibility of employing these cell systems to examine the biological bases and the mechanism of MCPyV infection in both malignant and non-malignant contexts across the respiratory tract.

Once we assessed the capability of respiratory cell lines to sustain viral replication and transcription, the DDR pathway was explored. In detail, our study targeted a panel of established DDR markers previously reported as modulated by MCPyV [13], thereby capturing key pathway dynamics.

Similar to our previous research, where higher levels of DDR transcripts were reported in MCPyV-positive MCC, an overexpression of DDR components, including *ATM*, *ATR*, *Chk1*, *Chk2*, *H2AX*, *p53*, *p21*, and *Rad51*, was observed in infected cells [13].

Notably, virus-mediated alteration of DDR gene transcription could represent an early event that plausibly contributes to sustained DDR, which has so far been mainly attributed to post-translational modification [9,26].

Specifically, the early upregulation of genes encoding for key DDR sensors (*ATM*, *ATR*) and checkpoint kinases suggests that host cells rapidly detect viral DNA as a source of genomic stress, leading to the activation of canonical signaling cascades, as supported by the modulation of downstream effectors such as *H2AX*, *p53* and *p21* [11,27]. Moreover, the overexpression of *Rad51* may indicate the recruitment of homologous recombination (HR). These findings suggest that MCPyV primarily exploits HR for DNA repair across respiratory models, with non-homologous end joining (NHEJ) playing a minor role, thus modulating the DDR pathway at multiple levels [28].

An interesting finding was that, despite the lack of functional p53 in H1299 cells, *p53* transcripts were detectable and modulated by MCPyV infection. In addition, since *p21* was also found overexpressed in infected cells, a p53-independent mechanism may occur in this cell system, suggesting that multiple regulatory mechanisms may contribute to its modulation during viral infection [29].

It is plausible that MCPyV exploits this early DDR activation to create a cellular environment beneficial for its own replication [12,30,31]. Indeed, several DNA viruses are recognized to manipulate host DDR to favor viral genome replication [7]. However, the disruption of normal mechanisms involved in DNA repair may promote genomic instability and tumorigenesis [31]. Specifically, previous studies suggested that the host DDR alteration by MCPyV may be relevant for virus-driven tumorigenesis, leading to abnormal viral DNA replication, integration and oncogenic progression [12,13]. Therefore, while early DDR activation may support viral replication, the persistent perturbation of this pathway may be involved in cellular transformation.

Interestingly, at later time-points (8 d.p.i.), DDR transcripts’ levels mainly returned to baseline, particularly in tumoral cells. This may reflect a transient nature of DDR induction, whereby the virus actively attenuates prolonged DDR signaling to prevent host cell death or detection by antiviral mechanisms [32]. Moreover, based on previous studies which reported a decline of DDR transcripts associated with advanced tumor status [26], it is possible to speculate that long-term viral persistence may attenuate DDR activity and contribute to tumor progression [33]. In non-malignant cells, instead, some factors remained significantly overexpressed even at late stages of viral infection. Particularly, in ALI cultures, *H2AX* revealed a peak in gene expression at 8 d.p.i., suggesting a delayed accumulation of DNA damage. In this framework, further studies aimed at investigating later time-points of viral infection will help to clarify whether MCPyV persistence may be involved in lung cancer progression and will contribute to a better understanding of DDR dynamics during MCPyV infection.

Differences in viral loads and DDR modulation across cell models highlight the importance of cellular contexts, including differentiation and transformation status, that may significantly affect host responses to viral infection. Exploring the mechanisms underlying these differences may be relevant for understanding MCPyV tropism, persistence and pathogenesis in the respiratory tract.

Overall, these results suggest a viral contribution in manipulating DDR cascade at the RNA level and support gene expression as indicative of DNA repair [34,35] and therefore as possible candidate biomarkers of damage and tumor progression.

Notably, despite providing new insights about MCPyV-mediated DDR modulation in respiratory models, this study has some limitations. Indeed, although monitoring mRNA levels of DDR components could support the role of the virus in triggering this signaling pathway, integrating these findings with RNA-seq for global gene expression, as well as protein expression and phosphorylation pattern analysis, will offer a more comprehensive overview of virus-mediated DDR activation. In this context, it can be hypothesized that the upregulation of DDR genes in infected cells may correlate with an increased expression and phosphorylation of key DDR factors, including ATM (Ser1981), Chk2 (Thr68), Chk1 (Ser345), p53 (Ser15/20), and H2AX (γH2AX), suggesting virus-induced DDR signaling [11,12,36]. Notably, the observed overexpression of p21 could reflect p53 activation, potentially mediated by phosphorylation-dependent stabilization.

## 4. Materials and Methods

### 4.1. Cell Cultures

HBEC-KT (Evercyte GmbH, Vienna, Austria) cells were grown in Keratinocyte SFM medium (Life Techonologies Inc., Carlsbad, CA), whereas the NSCLC cell lines, A549 cells and H1299 cells (ATCC, Manassas, VA, USA) were grown in Dulbecco’s modified Eagle’s medium (DMEM) and RPMI medium, respectively, supplemented with 100 U of penicillin and 100 µL of streptomycin per mL (Sigma-Aldrich S.r.l., Milano, Italia) and fetal bovine serum (FBS) (10%). The cells were incubated at 37 °C in the presence of 5% CO_2_ and propagated at a ratio of 1:4. The cells were then seeded in 12-well plates and grown for 24 h in complete growth medium to reach 50–70% confluence on the day of transfection. 2D ALI cells were obtained as previously described [37]. The day before transfection, transwell inserts were washed twice, and the growth medium was replaced in the basal chamber.

### 4.2. Plasmids and Transfection Methods

MCPyV DNA was recovered using BamHI-digested pMCV-R17, a plasmid containing the entire genome of MCPyV prototype strain MCC350 (Addgene plasmid #24729, Watertown, MA, USA). The obtained viral linear DNA was gel-extracted, purified using a GenepHlow TM DNA Cleanup Maxi Kit (Geneaid Biotech Ltd., New Taipei City, Taiwan) and then quantified. A total of 2.5 µg of MCC350 strain DNA was used to transfect cells (10^5^), following the specifications of the Xfect TM Transfection Reagent kit (Clontech Laboratories, Inc., Mountain View, CA, USA). Cells were incubated at 37 °C with the transfection mixture and, after 4 h, were washed with Dulbecco phosphate-buffered saline (PBS). Subsequently, cells were incubated with complete culture medium for the time course experiment. SPNTs were collected from monolayer cell cultures at 2, 4 and 8 days post-transfection (d.p.t.) and subjected to 6 cycles of freezing and thawing, followed by centrifugation at 2000 rpm for 5 min [19]. In order to avoid the use of different viral DNA copies in the infection experiments, the resulting clarified SPNTs were quantified through TaqMan-based quantitative polymerase chain reaction (qPCR), employing primers and probes for MCPyV sT, as previously described [38].

Transfection experiments were carried out even on ALI cells. At 2, 4 and 8 d.p.t., 200 µL of PBS was added to the apical surface and harvested after 10 min at 37 °C, before being collected for qPCR analysis.

### 4.3. Infection Experiments

Virions corresponding to 1 × 10^3^ copies per milliliter (copies/mL) were selected to infect freshly seeded A549, H1299 and HBEC-KT cells. After adsorption for 3 h, the cells were washed 3 times with PBS and incubated with complete medium for the time course experiment. Cells and SPNTs were collected at established time-points (2, 4, 8 days post-infection (d.p.i.)). For the infection of 2D ALI, cells were washed twice with PBS to remove the mucus. SPNTs obtained from HBEC-KT infections and corresponding to 1 × 10^3^ copies/mL were used to infect the apical side of the bronchial epithelium. Following 3 h, cells were washed 3 times with PBS and incubated. Therefore, apical PBS wash samples, basal medium and cells were collected at different sampling periods.

In parallel, non-infected control cells received the same manipulation as infected samples, without viral inoculum.

### 4.4. DNA Extraction and MCPyV Quantification by qPCR

Total DNA was extracted from cells using the Quick DNA Miniprep kit (Zymo Research, Irvine, CA, USA), following the manufacturer’s instruction. SPNTs were subjected to six cycles of freezing and thawing and then centrifuged at 2000 rpm for 10 min. The resulting clarified SPNT was directly used in molecular biology assays. The presence and quantity of MCPyV DNA were detected through qPCR using primers and probes targeting MCPyV sT [38]. Each sample was analyzed in triplicate, and viral loads were given as the mean of at least three positive reactions. Standard precautions designed to prevent contamination were followed, and a negative control was included in each run. Viral DNA was quantified using a standard curve consisting of serial dilutions of a plasmid containing the entire MCPyV genome with a known titer (range, 10^5^−10 copies/mL). The amount of cellular DNA was quantified simultaneously using an SYBR GREEN PCR for the house-keeping *GAPDH* gene and used to normalize the MCPyV DNA [19]. The data were expressed as copies of viral DNA per cell based on DNA content (copies/cell) for the cells and as copies of viral DNA per milliliter (copies/mL) for the SPNTs.

### 4.5. RNA Isolation and cDNA Synthesis

Total RNA was extracted from cells using the Total RNA Purification Kit (Norgen, Thorold, ON, Canada). After the RNA quality and quantity assessment by A230/A260 ratios, cDNA was generated through reverse transcription using the SensiFAST cDNA Synthesis kit (Meridian Bioscience, Cincinnati, OH, USA).

### 4.6. Relative Quantification of Transcript Levels by RT-qPCR

An aliquot of cDNA was used for qPCR analysis to examine MCPyV *LTAg* and *VP1* gene expression [22]. RNA levels were normalized to the *GAPDH* gene, and relative expression was calculated employing the ΔCt method and expressed as 2^−ΔCt^. Moreover, quantitative analysis of mRNA expression levels for *ATM*, *ATR*, *Chk1*, *Chk2*, *H2AX*, *p53*, *p21* and *Rad51* was performed using qPCR, as previously described [13,34,39,40]. The housekeeping gene *GAPDH* was used as an internal control to normalize target mRNA levels. Therefore, the relative expression of the genes was expressed as 2^−ΔΔCt^.

### 4.7. Statistical Analysis

A total of three independent experiments were performed, and data were reported as means ± standard deviation (SD). Normal distribution was defined with the Shapiro–Wilk test, and statistical significance was examined through paired Student *t*-tests with Bonferroni correction, using GraphPad Prism 10 software (version 9.2.0). *p* < 0.05 was considered statistically significant.

## 5. Conclusions

In conclusion, here we provide in vitro models for studying the interplay between MCPyV and host DDR within malignant and non-malignant contexts across the respiratory tract. While the precise mechanism remains to be fully elucidated, these preliminary findings support the role of MCPyV in manipulating the DDR pathway and represent a good starting point for delving into the relevance of DDR activation in virus-driven malignancies, thus contributing to the development of preventive strategies and better targeted therapeutic approaches.

## Figures and Tables

**Table 1:** MCPyV replication and transcription in respiratory cell lines.

	**Transfection Experiment**					
**Cell Line**	**Viral DNA Replication**					
	**Cell (Copies/Cell)**			**Spnts (Copies/mL)**		
	**2 d.p.i.**	**4 d.p.i.**	**8 d.p.i.**	**2 d.p.i.**	**4 d.p.i.**	**8 d.p.i.**
A549	5.3 × 10	7.2 × 10	8.4 × 10	3.2 × 10^2^	6.3 × 10^2^	1 × 10^3^
H1299	6.65 × 10	92.9 × 10	1.12 × 10^2^	5 × 10^2^	9.4 × 10^2^	1.15 × 10^3^
HBEC-KT	7.4 × 10	9.9 × 10	1.3 × 10^2^	5.8 × 10^2^	9.7 × 10^2^	1.2 × 10^3^
ALI	5.8 × 10	7.6 × 10	9.35 × 10	-	-	-
	**Infection Experiment**					
**Cell Line**	**Viral DNA Replication**					
	**Cell (Copies/Cell)**			**Spnts (Copies/mL)**		
	**2 d.p.i.**	**4 d.p.i.**	**8 d.p.i.**	**2 d.p.i.**	**4 d.p.i.**	**8 d.p.i.**
A549	1.2 × 10^2^	1.6 × 10^2^	1.75 × 10^2^	1 × 10^3^	1.5 × 10^3^	1.8 × 10^3^
H1299	1.9 × 10^2^	2.4 × 10^2^	2.7 × 10^2^	1.21 × 10^3^	1.5 × 10^3^	1.9 × 10^3^
HBEC-KT	2 × 10^2^	2.6 × 10^2^	2.9 × 10^2^	1.35 × 10^3^	1.8 × 10^3^	2 × 10^3^
ALI	1.25 × 10^2^	1.6 × 10^2^	1.9 × 10^2^	-	-	-
	**Viral Transcription**					
	**LTAg**			**VP1**		
	**2 d.p.i.**	**4 d.p.i.**	**8 d.p.i.**	**2 d.p.i.**	**4 d.p.i.**	**8 d.p.i.**
A549	0.01	0.018	0.027	0.46	0.68	0.96
H1299	0.015	0.031	0.04	0.53	0.88	1.02
HBEC-KT	0.023	0.046	0.3	0.65	0.89	1.15
ALI	0.015	0.026	0.21	0.3	0.47	0.64

## Data Availability

The original contributions presented in this study are included in the article material. Further inquiries can be directed to the corresponding authors.
